# Clinical characteristics and risk factors for right-sided infective endocarditis in Korea: a 12-year retrospective cohort study

**DOI:** 10.1038/s41598-024-60638-x

**Published:** 2024-05-07

**Authors:** Yongseop Lee, Jung Ho Kim, Jung Ah Lee, Sang Min Ahn, Min Han, Jin Young Ahn, Su Jin Jeong, Jun Yong Choi, Joon-Sup Yeom, Seung Hyun Lee, Nam Su Ku

**Affiliations:** 1 Department of Internal Medicine and AIDS Research Institute, Severance Hospital, Yonsei University College of Medicine, Seoul, 03722 South Korea; 2 Department of Thoracic and Cardiovascular Surgery, Severance Cardiovascular Hospital, Yonsei University College of Medicine, Seoul, 03722 South Korea

**Keywords:** Infectious diseases, Risk factors

## Abstract

Right-sided infective endocarditis (RSIE) is less common than left-sided infective endocarditis (LSIE) and exhibits distinct epidemiological, clinical, and microbiological characteristics. Previous studies have focused primarily on RSIE in patients with intravenous drug use. We investigated the characteristics and risk factors for RSIE in an area where intravenous drug use is uncommon. A retrospective cohort study was conducted at a tertiary hospital in South Korea. Patients diagnosed with infective endocarditis between November 2005 and August 2017 were categorized into LSIE and RSIE groups. Of the 406 patients, 365 (89.9%) had LSIE and 41 (10.1%) had RSIE. The mortality rates were 31.7% in the RSIE group and 31.5% in the LSIE group (*P* = 0.860). Patients with RSIE had a higher prevalence of infection with *Staphylococcus aureus* (29.3% vs. 13.7%, *P* = 0.016), coagulase-negative staphylococci (17.1% vs. 6.0%, *P* = 0.022), and gram-negative bacilli other than HACEK (12.2% vs. 2.2%, *P* = 0.003). Younger age (adjusted odds ratio [aOR] 0.97, 95% confidence interval [CI] 0.95–0.99, *P* = 0.006), implanted cardiac devices (aOR 37.75, 95% CI 11.63–141.64, *P* ≤ 0.001), and central venous catheterization  (aOR 4.25, 95%  CI 1.14–15.55, *P* = 0.029) were independent risk factors for RSIE. Treatment strategies that consider the epidemiologic and microbiologic characteristics of RSIE are warranted.

## Introduction

Right-sided infective endocarditis (RSIE) constitutes approximately 5–10% of all cases of infective endocarditis (IE) and has distinct epidemiologic, clinical, and microbiological characteristics compared with those of left-sided infective endocarditis (LSIE)^1–3^. However, most previous studies of RSIE have focused on patients with intravenous drug use (IVDU), and limited information is available on the characteristics of RSIE in populations in which IVDU is uncommon^2,4^.

A study conducted in South Korea, where IVDU is not prevalent, revealed differences in the clinical features of RSIE compared with studies conducted in countries with a higher prevalence of IVDU^5^. These differences were attributed to a lower proportion of *Staphylococcus aureus*, and patients with RSIE and LSIE had similar mortality rates^6–8^. Risk factors for RSIE include central venous catheterization, intracardiac devices, congenital heart disease, and IVDU^2^.

As the population ages and underlying diseases increase, the number of patients with these risk factors is expected to rise. However, few studies have examined risk factors for RSIE in populations in which IVDU is uncommon. Therefore, our study aimed to investigate the clinical characteristics and risk factors of RSIE in South Korea, where IVDU is infrequent.

## Methods

### Study population

We retrospectively analyzed adult patients with IE who were admitted to Severance Hospital, a large tertiary care teaching hospital with 2,400 beds in South Korea, between November 2005 and August 2017. Patients with IE involving both sides were excluded from the study. The study received approval from the Institutional Review Board of Yonsei University Health System Clinical Trial Center (4–2018-0248), and the need for patient consent was waived due to the retrospective nature of the study. All methods were performed in accordance with the relevant guideline (STROBE checklist) and regulations.

IE was defined as definite or possible based on the Duke criteria, which were modified in 2000, and patients meeting these criteria were included in the study^9^. A multidisciplinary team comprising cardiologists, cardiovascular surgeons, and infectious disease specialists managed patients admitted for suspicion of IE. Surgery was performed in accordance with the guidelines of the American Heart Association and South Korea's national guidelines^10,11^. The appropriate surgical approach was determined through agreement among cardiologists, cardiovascular surgeons, and the advice of infectious disease specialists. Transesophageal echocardiography was conducted in most patients, including those with negative transthoracic echocardiographic findings.

Follow-up visits to the outpatient clinic were scheduled at 1 week and at 1, 3, 6, and 12 months after discharge. During each visit, patients underwent a systematic review and physical examination to assess evidence of heart failure and IE relapse. At the 6-month follow-up, echocardiography was performed to evaluate valvular and ventricular function. Subsequently, follow-up visits to the outpatient clinic were conducted every 6 months.

### Variables and definitions

Nosocomial infection was defined as an infection occurring more than 48 h after hospitalization, with no evidence of infection at admission. It was also diagnosed if IE occurred within 60 days after hospital discharge when a high-risk procedure for bacteremia was performed or when any predisposing factor for IE was present during hospitalization, including dental manipulation, gastrointestinal manipulation, gynecological procedures, urological manipulation, and invasive intravascular techniques (intravascular device implantation, pacemaker insertion, and cardiac catheterization)^12–14^. Cardiac devices were defined as implantable pacemakers or defibrillators^15^.

The Charlson Comorbidity Index was used to categorize patients based on comorbidities identified on hospital admission^16^. The Sequential Organ Failure Assessment was used to stratify disease severity. Causative microorganisms were defined as those present in blood or tissue samples (valves and/or vegetation).

Mortality referred to all-cause mortality. Mortality data were obtained from the Ministry of the Interior and Safety of South Korea, which compiles mortality records of all Korean citizens. Mortality data were collected through September 2019.

### Microbiological tests

Identification testing was performed using the VITEK 2 automated analyzer system (bioMérieux, Marcy-l’Étoile, France) until 2013. From 2013 onward, the Bruker Biotyper matrix-assisted laser desorption ionization-time of flight mass spectrometry (MALDI-TOF MS) system was used for identification testing (Bruker Daltonics, Bremen, Germany). Susceptibility tests were performed using the VITEK 2 automated analyzer system with a VITEK AST2 N212 card (bioMérieux). The results of antibiotic susceptibility testing were interpreted using the criteria of the Clinical and Laboratory Standards Institute^17^.

### Statistical analysis

We evaluated the clinical and microbiological characteristics of LSIE and RSIE. Continuous variables were compared using the independent samples* t*-test or Mann–Whitney* U*-test, depending on the normality of distribution. Pearson’s chi-squared test was used to compare two categorical variables, and Fisher's exact test was used when the expected value of at least one cell was < 5. Kaplan–Meier survival analysis was performed to assess the long-term prognosis. Variables with a *P*-value of < 0.05 in the univariable analysis were included in the multivariable logistic regression model to identify independent risk factors for RSIE. The variance inflation factor (VIF) was used to assess multicollinearity, with a VIF value greater than 10 indicating multicollinearity. The goodness-of-fit was tested using the Hosmer–Lemeshow test. *P*-values < 0.05 were considered statistically significant. All statistical analyses were performed using R V.4.2.2 (The R Foundation for Statistical Computing, Vienna, Austria).

### Ethics approval and informed consent statement

This study was approved by the Institutional Review Board of Yonsei University Health System Clinical Trial Center (4–2018-0248). The requirement for patient consent was waived owing to the retrospective nature of the study.

## Results

### Study population and characteristics

**Figure 1:**
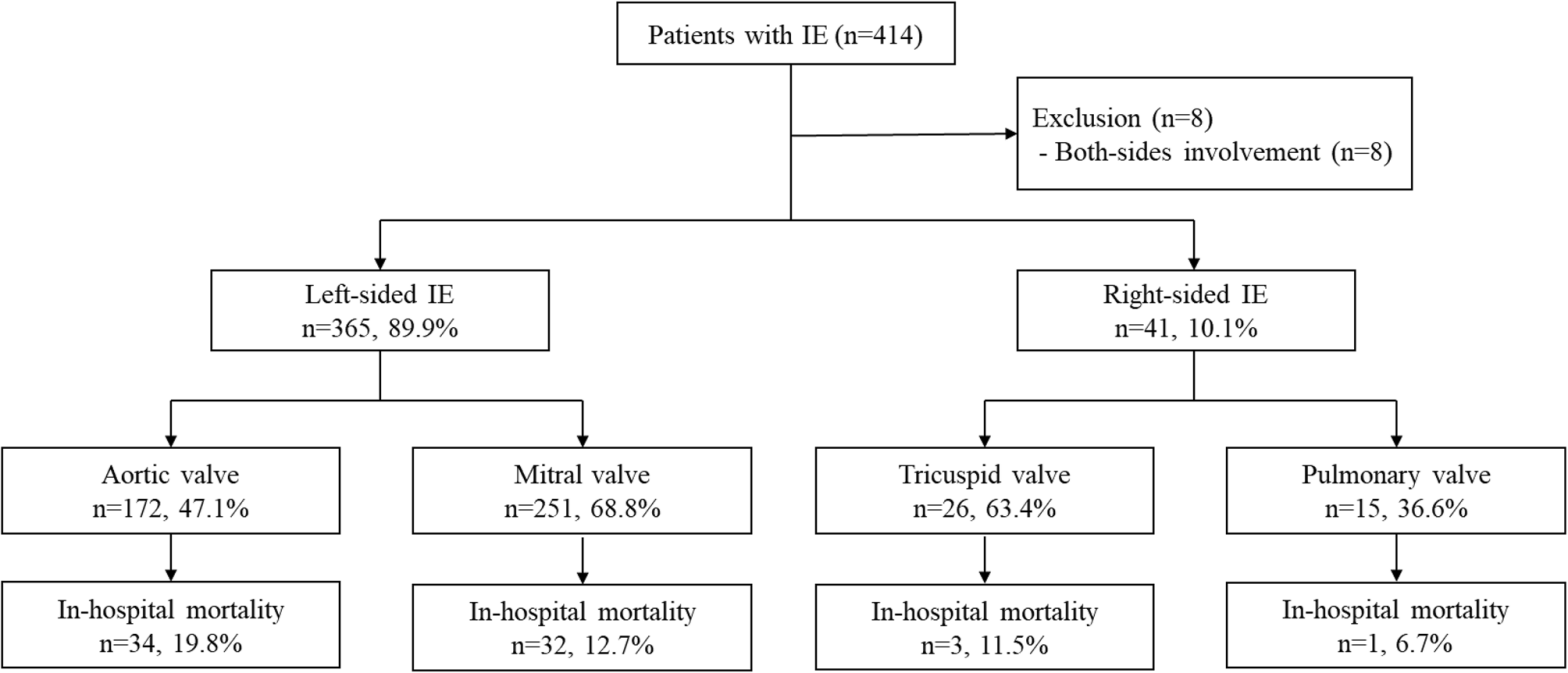
Flowchart of the selection of patients with infective endocarditis during the study period.

A total of 414 patients who met the inclusion criteria were initially enrolled in the study (Fig. 1). Of these patients, eight with involvement of both sides were excluded. The final analysis included 406 patients, of whom 365 and 41 were diagnosed with LSIE and RSIE, respectively.

The median age of patients with RSIE was 49 years (interquartile range [IQR]: 32–66 years), whereas that of patients with LSIE was 57 years (IQR: 44–68 years) (Table 1). Sex distribution did not differ significantly between the two groups (men: 67.1% vs. 53.7%, *P* = 0.121). A history of previous valve surgery was more prevalent in the RSIE group than in the LSIE group (18.1% vs. 34.1%, *P* = 0.025). The number of patients who had undergone cardiac device implantation was also higher in the RSIE group than in the LSIE group (1.4% vs. 31.7%, *P* < 0.001).

**Table 1:** Clinical characteristics of patients with infective endocarditis.

	Number (%)		
	Left-sided IE (*n* = 365)	Right-sided IE (*n* = 41)	*P*-value
Age	57.0 [44.0–68.0]	49.0 [32.0–66.0]	0.057
Men	245 (67.1%)	22 (53.7%)	0.121
Definite infective endocarditis^a^	288 (78.9%)	26 (63.4%)	0.040
Possible infective endocarditis^a^	77 (21.1%)	15 (36.6%)	0.040
Nosocomial infection	74 (20.3%)	12 (29.3%)	0.256
Type of infective endocarditis			
Native valve	312 (85.5%)	33 (80.5%)	0.537
Prosthetic valve	53 (14.5%)	8 (19.5%)	0.537
CIED	0 (0.0%)	3 (7.3%)	< 0.001
Comorbidities			
Previous valve surgery	66 (18.1%)	14 (34.1%)	0.025
Implanted cardiac devices^b^	5 (1.4%)	13 (31.7%)	< 0.001
Previous infective endocarditis	17 (4.7%)	2 (4.9%)	1.000
Immunosuppressive therapy	13 (3.6%)	4 (9.8%)	0.143
Chemotherapy within 30 days	11 (3.0%)	3 (7.3%)	0.327
Antibiotic treatment within 30 days	57 (15.6%)	13 (31.7%)	0.018
Central venous access	21 (5.8%)	7 (17.1%)	0.017
Diabetes mellitus	67 (18.4%)	7 (17.1%)	1.000
Congestive heart failure	24 (6.6%)	5 (12.2%)	0.315
Renal disease	36 (9.9%)	5 (12.2%)	0.844
Liver disease	24 (6.6%)	3 (7.3%)	1.000
Hemodialysis	21 (5.8%)	1 (2.4%)	0.600
Cancer	45 (12.3%)	8 (19.5%)	0.294
Connective tissue disease	12 (3.3%)	1 (2.4%)	1.000
Charlson comorbidity index	2.0 [0.0–4.0]	1.0 [0.0–6.0]	0.515
Severity index			
SOFA score	1.0 [1.0–3.0]	1.0 [1.0–3.0]	0.809
Affected valve			
Aortic valve	172 (47.1%)	–	–
Mitral valve	251 (68.8%)	–	–
Tricuspid valve	–	26 (63.4%)	–
Pulmonary valve	–	15 (36.6%)	–
Echocardiographic findings			
TTE performed	360 (98.6%)	39 (95.1%)	0.316
TEE performed	268 (73.4%)	18 (43.9%)	< 0.001
Associated ventricular septal defect	6 (1.6%)	3 (7.3%)	0.075
Large vegetation (> 2 cm)	37 (10.1%)	9 (22.0%)	0.045

CIED, cardiovascular implantable electronic device; TTE, transthoracic echocardiography; TEE, transesophageal echocardiography.

^a^Definite and possible infective endocarditis were defined according to the Duke criteria.

^b^Implanted cardiac devices were defined as implantable pacemakers or defibrillators.

Among the patients with RSIE, 26 (63.4%) had tricuspid valve involvement, whereas 15 (36.6%) had pulmonary valve involvement. Transthoracic echocardiography was performed in most of the patients, with no difference in frequency between those with RSIE and LSIE. Transesophageal echocardiography was performed more frequently in patients with LSIE than in those with RSIE (73.4% of LSIE cases and 43.9% of RSIE cases; *P* < 0.001). The presence of large vegetations, > 2 cm in diameter, was more commonly observed in the RSIE group than in the LSIE group (22.0% vs. 10.1%, *P* = 0.045).

Patients with RSIE involving the pulmonary valve and those with RSIE involving the tricuspid valve were compared (Supplementary Table S1). Patients with pulmonary valve IE were younger than those with tricuspid valve IE (44 vs. 58 years, *P* = 0.010). In the pulmonary valve IE group, a higher proportion of patients had predisposing factors for IE, including 11 (73.3%) with a history of valve surgery and 4 (26.7%) with cardiac devices. In the tricuspid valve IE group, only 3 patients (11.5%) had a history of undergoing valve surgery. Of the 15 pulmonary valve IE cases, 13 (86.7%) had congenital heart disease, including patent ductus arteriosus, tetralogy of Fallot, pulmonary atresia, and ventricular septal defect.

### Clinical outcomes

In the RSIE group, 21 patients (51.2%) underwent surgery; in the LSIE group, 243 patients (66.6%) underwent surgical intervention (*P* = 0.075). In the RSIE group, embolism prevention was the most common surgical indication (*n* = 13; 31.7%), followed by heart failure (*n* = 12; 29.3%). Heart failure (65.8% vs. 29.3%, *P* < 0.001) and paravalvular complications (17.3% vs. 0.0%, *P* = 0.008), as surgical indications, were observed more frequently in the LSIE group than in the RSIE group.

**Figure 2:**
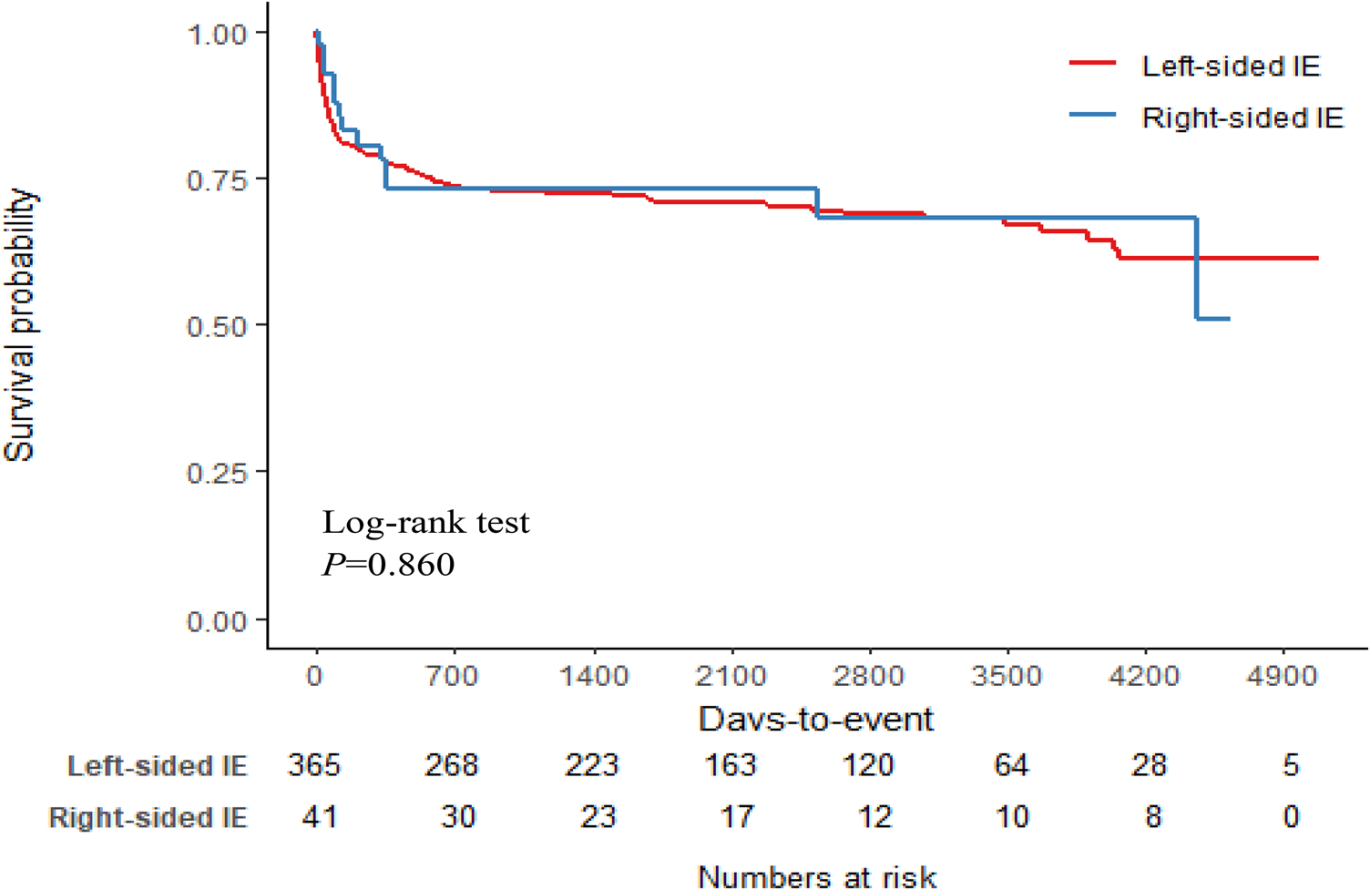
Kaplan–Meier curves of the overall survival rates of patients with infective endocarditis IE, infective endocarditis.

Overall, the outcomes of the two groups were similar (Table 2). In the RSIE group, four patients (9.8%) died during their hospital stay, which did not differ significantly from the mortality rate in the LSIE group (*n* = 53, 14.5%, *P* = 0.551). The overall mortality rates were 31.7% in patients with RSIE and 31.5% in patients with LSIE. Kaplan–Meier curves showed that the overall survival rates of the two groups did not differ significantly (*P* = 0.860, log-rank test) (Fig. 2). The median follow-up duration was 1,829 days (IQR: 558.75–3104.25 days).

**Table 2:** Outcomes of patients with infective endocarditis.

	Number (%)		
	Left-sided IE (*n* = 365)	Right-sided IE (*n* = 41)	*P*-value
Surgery performed	243 (66.6%)	21 (51.2%)	0.075
Surgery indication			
Heart failure	240 (65.8%)	12 (29.3%)	< 0.001
Paravalvular complications^a^	63 (17.3%)	0 (0.0%)	0.008
Uncontrolled infection	32 (8.8%)	6 (14.6%)	0.347
Pacemaker infection	1 (0.3%)	7 (17.1%)	< 0.001
Prevention of embolism	121 (33.2%)	13 (31.7%)	0.991
New onset heart failure	55 (15.1%)	2 (4.9%)	0.123
New conduction abnormality	29 (7.9%)	1 (2.4%)	0.336
Renal failure	51 (14.0%)	7 (17.1%)	0.762
Systemic emboli excluding CNS	27 (7.4%)	4 (9.8%)	0.819
In-hospital mortality	53 (14.5%)	4 (9.8%)	0.551
28-day mortality	32 (8.8%)	1 (2.4%)	0.269
1-year mortality	84 (23.0%)	11 (26.8%)	0.724
Overall mortality	115 (31.5%)	13 (31.7%)	1.000

IE, infective endocarditis; CNS, central nervous system.

^a^Paravalvular complication means locally uncontrolled infection (abscess, false aneurysm, and fistula).

### Microbiological characteristics

Microbiological analyses revealed several differences in the distribution of microorganisms between the LSIE and RSIE groups. Patients with RSIE showed higher prevalence of infection with *S. aureus* (LSIE: 13.7% vs. RSIE: 29.3%, *P* = 0.016), coagulase-negative staphylococci (CoNS) (LSIE: 6.0% vs. RSIE: 17.1%, *P* = 0.022), and gram-negative bacilli (GNB) other than HACEK (*Haemophilus parainfluenzae, Haemophilus aphrophilus*, *Actinobacillus actinomycetemcomitans*, *Cardiobacterium hominis*, *Eikenella corrodens*, and *Kingella kingae*) (LSIE: 2.2% vs. RSIE: 12.2%, P = 0.003) (Table 3). In contrast, streptococcal infections were more common in the LSIE group than in the RSIE group (LSIE: 38.1% vs. RSIE: 14.6%, *P* = 0.005).

**Table 3:** Microbiology of infective endocarditis.

	Number (%)		
	Left-sided IE (*n* = 365)	Right-sided IE (*n* = 41)	*P*-value
*S. aureus*	50 (13.7%)	12 (29.3%)	0.016
Methicillin sensitive	29 (7.9%)	7 (17.1%)	0.097
Methicillin resistant	21 (5.8%)	5 (12.2%)	0.207
Coagulase-negative staphylococci	22 (6.0%)	7 (17.1%)	0.022
Methicillin sensitive	10 (2.7%)	2 (4.9%)	0.779
Methicillin resistant	14 (3.8%)	5 (12.2%)	0.044
Enterococci	32 (8.8%)	0 (0.0%)	0.095
Streptococci	139 (38.1%)	6 (14.6%)	0.005
HACEK	2 (0.5%)	0 (0.0%)	1.000
GNB except HACEK	8 (2.2%)	5 (12.2%)	0.003
*Candida* spp.	3 (0.8%)	0 (0.0%)	1.000
Others	8 (2.2%)	0 (0.0%)	0.715

IE, infective endocarditis; GNB, gram-negative bacilli; HACEK, *Haemophilus parainfluenzae, Hemophilus aphrophilus*, *Actinobacillus actinomycetemcomitans*, *Cardiobacterium hominis*, *Eikenella corrodens*, and *Kingella kingae.*

Methicillin-resistant staphylococci were more common in patients with RSIE. Methicillin-resistant *S. aureus* (MRSA) and methicillin-resistant CoNS were found in 12.2% of patients with RSIE, and 5.8% and 3.8% of patients with LSIE, respectively. Methicillin resistance was observed in 41.7% of patients with RSIE and 42.0% of patients with LSIE infected with *S. aureus*, and in 71.4% of patients with RSIE and 63.6% of patients with LSIE infected with CoNS. Overall, methicillin-resistant staphylococci accounted for 24.4% and 9.6% of all cases of RSIE and LSIE, respectively.

### Risk factors for right-sided infective endocarditis

Univariate analysis revealed that younger age, history of valve surgery, implanted cardiac devices, recent antibiotic use, and central venous catheterization were associated with RSIE (Table 4). The multivariable model identified that RSIE was independently associated with age (aOR: 0.97, 95% CI: 0.95–0.99, *P* = 0.006), implanted cardiac devices (aOR: 37.75, 95% CI: 11.63–141.64; *P* < 0.001), and central venous catheters (aOR: 4.25, 95% CI: 1.14–15.55, *P* = 0.029; Table 4).

**Table 4:** Risk factors of right-sided infective endocarditis.

	Univariable analysis		Multivariable analysis	
	OR (95% CI)	*P*-value	aOR (95% CI)	*P*-value
Age	0.98 (0.96–1.00)	0.036	0.97 (0.95–0.99)	0.006
Male sex	0.57 (0.30–1.10)	0.088	–	–
Previous valve surgery	2.35 (1.14–4.66)	0.017	1.17 (0.44–2.79)	0.737
Cardiac devices	33.43 (11.72–110.55)	< 0.001	37.75 (11.63–141.64)	< 0.001
Recent antibiotic use^a^	2.51 (1.19–5.05)	0.012	1.68 (0.54–4.71)	0.344
Central venous access	3.37 (1.25–8.19)	0.01	4.25 (1.14–15.55)	0.029

aOR, adjusted odds ratio; IE, infective endocarditis; OR, odds ratio.

^a^Recent administration of antibiotics is defined as the administration of agents within the past 30 days.

*Hosmer-Lemshow goodness of fitness:* P* = 0.325.

## Discussion

This study revealed the distinct bacterial causes and clinical manifestations of RSIE and LSIE. Patients with RSIE are more likely to have predisposing factors such as a history of valve surgery, implanted cardiac devices, and central venous catheter placement. Patients with RSIE also tended to have larger vegetations and a higher prevalence of infection with *S. aureus*, CoNS, and GNB other than HACEK, compared with patients with LSIE. The risk of death did not differ significantly between the two groups. The risk factors for RSIE were younger age, implanted cardiac devices, and central venous catheterization.

The risk factors for RSIE identified in this study align with those reported in previous studies, although the presence of congenital heart disease was not investigated^2^. Currently, commonly used implantable cardiac devices include pacemakers, implantable cardioverter defibrillators, and cardiac resynchronization therapy implants^18^. The leads of these cardiac devices’ are primarily positioned in the right heart, and this anatomical arrangement appears to be associated with the occurrence of IE. In patients with pacemakers, approximately two-thirds show valvular involvement, with tricuspid valve involvement^19^. observed in about 60% of pacemaker-lead IE cases.

Central venous catheter insertion is also a common risk factor. The potential mechanism is believed to involve the abrasion of the tricuspid leaflet and endocardium because of the close proximity to the tricuspid valve and the forceful and rapid jet of injections through the catheter^20^. Several studies have compared the infection risk between central venous catheters and peripherally inserted central catheters (PICCs), with discrepancies in their findings^21–23^. According to a previous meta-analysis, the degree of infection risk associated with PICCs was found to be similar to that with central venous catheters in inpatient settings but lower in outpatient settings^24^.

In addition, young age was found to be a risk factor for RSIE in this study. This finding is attributable to the increased involvement of the pulmonary valve observed in patients with RSIE. The median age of patients with tricuspid valve involvement was 58 years, which was comparable to that of patients with LSIE (57 years). In patients with pulmonary valve IE, the median age was 44 years, which was significantly lower. Although previous studies on RSIE associated with IVDU have reported tricuspid valve involvement in a high percentage of patients and considered pulmonary valve involvement to be rare, this study showed a substantial proportion (36.6%) of patients with RSIE with pulmonary valve involvement^6,25,26^. This age difference in pulmonary valve IE and the higher incidence of pulmonary valve involvement probably contribute to the higher occurrence of RSIE at a younger age.

Regarding microbiology, this study showed a higher prevalence of *S. aureus*, CoNS, and GNB other than HACEK infections in RSIE compared with LSIE. *S. aureus* is the most common causative pathogen in RSIE, accounting for 60%–90% of cases^2^. In our study, *S. aureus* was the most common causative microorganism, constituting 29.3% of RSIE cases, which is substantially lower than the previous study. This discrepancy is likely attributable to the regional characteristics of the lower IVDU in our study population. When comparing our study with previous research conducted in the same region, the prevalence of *S. aureus* infections was similar^5^.

We also observed a relatively high prevalence of CoNS infection and a relatively low prevalence of streptococcal infection. These differences in microbiology are likely to be associated with the increased incidence of RSIE in patients with implanted cardiac devices and central catheters. Staphylococcal species are the major causative microorganisms of pacemaker infections, accounting for 70–95% of cases^27^. The most common causative organisms of catheter-related bloodstream infections are CoNS, *S. aureus*, *Candida* species, and enteric GNB^28^. In RSIEs not associated with IVDUs, the microbiology is related to that of pacemaker and catheter-related infections—infections associated with these conditions are common in RSIE.

Infections caused by non-HACEK GNBs are infrequent in IE. The International Collaboration on Endocarditis reported that only 1.8% of IE cases were non-HACEK GNB infections^29^. Similarly, in this study, infections caused by GNB accounted for only 2.2% of cases of LSIE. However, 12.2% of cases of RSIE were GNB infections, a significantly higher proportion than that in LSIE cases. The current guidelines for empiric treatment of IE do not differentiate between RSIE and LSIE and focus mainly on infections caused by gram-positive organisms^2^. Based on the results of this study, about 10% of RSIE patients may receive inappropriate empirical antibiotics if treated according to the guidelines. Therefore, further research is needed on risk factors and treatment strategies for GNB infection in RSIE, and further consideration of empirical treatment may be warranted based on the epidemiologic and microbiologic characteristics of RSIE.

Methicillin-resistant staphylococcal infections accounted for approximately 25% of all patients with RSIE. Among cases of staphylococcal infection, methicillin resistance was observed in 41.7% of patients with *S. aureus* infection and 71.4% of patients with CoNS infection. This finding indicates a notably high rate of methicillin resistance. Additionally, when considering nosocomial infections alone, methicillin resistance was observed in 58.3% of all patients with RSIE. Community-acquired infections exhibited a lower resistance rate, with 10.3% of the patients showing methicillin resistance. Therefore, methicillin resistance should be considered in RSIE, particularly in patients with nosocomial infections.

Furthermore, this study revealed a higher incidence of large vegetations (≥ 2 cm) in patients with RSIE, with 22% of patients with RSIE having large vegetations. A study conducted in the same region also reported a high prevalence of large vegetations (33.3%)^5^. In both studies, the incidence of large vegetations in LSIE was approximately 10%, which is significantly lower than the rates observed in patients with RSIE. In a study on IVDU-related IE, 15.3% of individuals with IE had large vegetations. This finding suggests that large vegetation is more common in patients with RSIE in regions where IVDU is less common^30^. Given the previous findings that large vegetation is a poor prognostic factor for in-hospital mortality in RSIE, identifying the presence of these large vegetation types in RSIE is important owing to its potential association with unfavorable patient outcomes^30^. Therefore, conducting further research to establish early diagnostic strategies for RSIE may help improve the patient’s prognosis.

This study also showed no significant difference in long-term mortality between the RSIE and LSIE groups. The overall mortality rates for LSIE and RSIE were 31.5% and 31.7%, respectively, with no significant difference. A study conducted in countries with low IVDU rates also found no difference in in-hospital mortality between the two groups, whereas a study on IVDU-related IE showed favorable long-term survival of patients with RSIE^5,31^. The median age of patients with IVDU-related IE was 39 years, significantly younger than those without non-IVDU-related IE^31^. However, in this study, the median age of patients with RSIE was 49 years, suggesting enrolment of older patients than the previous study. Additionally, there were no significant differences in comorbidities between the RSIE and LSIE groups, which may have contributed to the lack of significant differences in long-term outcomes. Therefore, we hypothesize that the better prognosis of RSIE compared to LSIE is a result of inter-study differences in patient age and comorbidities rather than the nature of the disease.

Several limitations should be considered when interpreting the results of this study. It was a retrospective cohort study conducted in a single large tertiary hospital in South Korea, potentially introducing selection bias and limiting its ability to fully represent the entire population of individuals with IE. Additionally, the small number of patients with RSIE may pose a challenge when interpreting the data. Detailed information on IVDU may not have been thoroughly investigated owing to the regional characteristics in which IVDU is less common.

Despite the limitations, our findings are meaningful because RSIE is rare in areas of low IVDU prevalence. One study has evaluated the characteristics of RSIE in areas of low IVDU prevalence, and it included 39 cases of RSIE^5^, a similar number of RSIE cases to this work. Considering the low incidence of RSIE, a multicenter prospective cohort study may be warranted to provide a more comprehensive description of the disease characteristics.

## Conclusion

In regions with a low IVDU prevalence, RSIE demonstrated a higher incidence of infection with *S. aureus*, CoNS, and GNB other than HACEK. Methicillin resistance can be considered in RSIE, particularly in patients who developed nosocomial infections. Overall mortality did not differ between the RSIE and LSIE groups. Younger age, implanted cardiac devices, and central venous catheterization were identified as the risk factors for RSIE. With the increased use of cardiac devices and central venous catheters, the increasing incidence of RSIE is a potential concern and treatment strategies that consider epidemiologic and microbiologic characteristics of RSIE is warranted.

### Supplementary Information

Supplementary Table S1.

## Data Availability

The datasets used for the current study are available from the corresponding author upon reasonable request.
