# Supplementary material for: Clinical characteristics and risk factors for right-sided infective endocarditis in Korea: a 12-year retrospective cohort study
Source: Sci Rep. 2024 May 7;14:10466. doi: 10.1038/s41598-024-60638-x (PMC11076501; doi:10.1038/s41598-024-60638-x)
Supplement: Supplementary file 1 — Supplementary Table S1. [file 41598_2024_60638_MOESM1_ESM.docx]

Supplementary Table S1. Clinical characteristics of patients with right-sided infective endocarditis (RSIE)

|  | Number (%) | | |
| --- | --- | --- | --- |
|  | Pulmonary valve  (n=15) | Tricuspid valve  (n=26) | *P*-value |
| Age | 44 [30.5–47] | 58 [47–71] | 0.010 |
| Male | 5 (33.3%) | 17 (65.4%) | 0.097 |
| Definite infective endocarditis^a^ | 8 (53.3%) | 18 (69.2%) | 0.496 |
| Possible infective endocarditis^a^ | 7 (46.7%) | 8 (30.8%) | 0.496 |
| Nosocomial | 1 (6.7%) | 11 (42.3%) | 0.039 |
| **Type of infective endocarditis** |  |  |  |
| Native valve | 7 (46.7%) | 26 (100.0%) | <0.001 |
| Prosthetic valve | 8 (53.3%) | 0 (0.0%) | <0.001 |
| CIED | 0 (0.0%) | 3 (11.5%) | 0.457 |
| **Comorbidities** |  |  |  |
| Previous valve surgery | 11 (73.3%) | 3 (11.5%) | <0.001 |
| Implanted cardiac devices^b^ | 4 (26.7%) | 9 (34.6%) | 0.858 |
| Previous infective endocarditis | 2 (13.3%) | 0 (0.0%) | 0.248 |
| Antibiotic treatment within 30 days | 3 (20.0%) | 10 (38.5%) | 0.381 |
| Central venous access | 1 (6.7%) | 6 (23.1%) | 0.361 |
| Charlson Comorbidity Index | 0.0 [ 0.0–1.0] | 2.0 [ 1.0–8.0] | 0.005 |
| **Severity index** |  |  |  |
| SOFA score | 1.0 [1.0–2.0] | 1.0 [1.0–3.0] | 0.502 |
| **Surgery performed** | 9 (60.0%) | 12 (46.2%) | 0.596 |
| **Surgery Indication** |  |  |  |
| Heart failure | 3 (20.0%) | 9 (34.6%) | 0.526 |
| Uncontrolled infection | 3 (20.0%) | 3 (11.5%) | 0.780 |
| Pacemaker infection | 0 (0.0%) | 7 (26.9%) | 0.076 |
| Prevention of embolism | 2 (13.3%) | 11 (42.3%) | 0.116 |

CIED: cardiovascular implantable electronic device; SOFA, Sequential Organ Failure Assessment
^a^Definite and possible infective endocarditis were defined according to the Duke criteria.
^b^Implanted cardiac devices were defined as implantable pacemakers or defibrillators.
